# Germline *BARD1* Mutation in High-Risk Chinese Breast and Ovarian Cancer Patients

**DOI:** 10.3390/cancers17152524

**Published:** 2025-07-30

**Authors:** Ava Kwong, Cecilia Y. S. Ho, Chun Hang Au, Edmond S. K. Ma

**Affiliations:** 1 Department of Surgery, The University of Hong Kong, Hong Kong SAR, China; 2 Breast Surgery Centre, Department of Surgery, Hong Kong Sanatorium & Hospital, Hong Kong SAR, China; 3 Cancer Genetics Centre, Hong Kong Sanatorium & Hospital, Hong Kong SAR, China; 4 Hong Kong Hereditary Breast Cancer Family Registry, Hong Kong SAR, China; 5 Division of Molecular Pathology, Department of Pathology, Hong Kong Sanatorium & Hospital, Hong Kong SAR, China

**Keywords:** germline, *BARD1*, Chinese, hereditary breast-ovarian cancer

## Abstract

Background: The prevalence of *BARD1* mutations in breast and ovarian cancers varies across different ethnic groups. Evaluating the cancer risk and clinical significance of *BARD1* mutations in the local Chinese patients with breast cancer, ovarian cancer, or both is clinically important for designing an appropriate surveillance scheme. Methods: This study used a 30 gene panel to identify *BARD1* germline mutations in 2658 breast and ovarian cancer patients. Results: Among this cohort, the *BARD1* mutation prevalence was 0.45% for breast cancer and 0.29% for ovarian cancer. In our 12 mutation carriers, we identified eight types of mutation variants, including three novel mutations. *BARD1* mutation carriers were more likely to have a family history of liver, prostate, and cervical cancers (*p*-values = 0.004, 0.018, and 0.037, respectively) than patients who tested negative for mutations. Among the *BARD1* mutants, the majority of the breast tumors were invasive ductal carcinoma (NOS type) (10/11, 90.9%) of high-grade disease (9/9, 100%) and half of them were triple-negative breast cancer (5/10, 50%). Conclusions: Although the prevalence of *BARD1* mutations is low and the penetrance is incomplete, we recommend including *BARD1* in the test panel for breast cancer patients. Our data suggest that more comprehensive surveillance management may be considered in mutation carriers due to the familial aggregation of a relatively wide spectrum of cancers.

## Simple Summary

This study explores the prevalence of *BARD1* mutations in breast and ovarian cancer among Chinese patients. *BARD1* mutations can vary across different ethnic groups, which is important for assessing cancer risk and developing effective monitoring strategies. This research involved a 30 gene panel and included 2658 patients. It found that *BARD1* mutations occurred in 0.45% of breast cancer cases and 0.29% of ovarian cancer cases. Among the 12 patients with *BARD1* mutations, eight different mutation types were identified, including three new variants. These mutation carriers were more likely to have family histories of other cancers, such as liver, prostate, and cervical cancers. Most breast tumors in mutation carriers were high-grade invasive ductal carcinoma, with a significant portion being triple-negative. Although *BARD1* mutations are rare, the findings suggest that testing for *BARD1* should be included in breast cancer panels, and mutation carriers may need closer monitoring due to associated family cancer histories.

## 1. Introduction

Breast cancer is the most common female cancer and ovarian is the sixth most prevalent cancer in Hong Kong. On average, 1 in 20 women worldwide will be diagnosed with breast cancer in their lifetime, while a woman getting ovarian cancer during her life is about 1 in 78. The risk for breast and ovarian cancers is further enhanced in hereditary breast–ovarian cancer (HBOC). HBOC is a well-studied cancer predisposition syndrome caused by germline loss-of-function mutations and pathogenic variants (PVs) in genes such as *BRCA1* or *BRCA2*. Multigene panel testing has revealed 10–20% HBOC-associated PVs [1,2]. However, further evidence and more conclusive cancer risk assessments are necessary before recommending surveillance management for mutation carriers in genes where PVs confer low or moderate penetrance effects. *BRCA1*-associated RING domain 1 (*BARD1*) is one of the genes considered to have low or moderate penetrance [3]. *BARD1* consists of a RING-finger domain at its N-terminal region, followed by three intervening ankyrin (ANK) repeat domains and two tandem *BRCA1* C-terminal domains (BRCT) [4]. *BARD1* shares a high degree of structural and functional homology with *BRCA1* within its BRCT and RING-finger domains, and these two proteins form a stable heterodimer [5]. *BARD1* has been shown to act as a tumor suppressor in a *BRCA1*-independent pathway [6] and is involved in the homologous recombination repair pathway [7], stabilizing the p53 tumor suppressor via its ANK and BRCT domains [8]. It interacts with the repeated sequences of the BCL3 ANK domains and modulates the activities of the transcription factor NF-κB in the TP53-dependent apoptotic signaling pathway [9]. Moreover, it also facilitates the ubiquitination of RNA polymerase II, thereby hindering the transcription of damaged DNA and preventing the ubiquitination of ER-alpha and ER-beta, which are involved in cellular proliferation during the development of breast cancer [10]. Additionally, a decrease in *BARD1* protein expression has been associated with cellular changes linked to a premalignant phenotype [11]. *BARD1* was shown to play a role in maintaining genomic integrity, and the loss of *BARD1* leads to chromosomal instability and embryonic death in the early stages [12]. On the other hand, various *BARD1* isoforms that lack functional domains, such as the RING-finger and ANK domains due to exon skipping, have been found to be upregulated in different cancers. Abnormal *BARD1* isoforms have been detected in non-small cell lung cancer (NSCLC), as well as in breast, colon, and ovarian cancers [13]. These isoforms are thought to have an oncogenic effect by interfering with the function of full-length *BARD1* and are believed to contribute to tumorigenesis and cancer progression [13,14].

The *BRCA1*-*BARD1* heterodimers are essential tumor suppressors in breast and ovarian cancers. These heterodimers also have additional functions in regulating the cell cycle, modulating the chromatin structure, and hormone signaling during cancer progression [8]. They are involved in DNA repair, replication fork protection, transcription, and tumor suppression [15]. In cancer cells, mutations that disrupt these heterodimers can lead to the detrimental degradation of both *BRCA1* and *BARD1* proteins [16].

Another important domain in *BARD1* is the BRCT domain. It facilitates the early recruitment of the *BRCA1*-*BARD1* heterodimer to DNA damage sites through a specific interaction with poly (ADP-ribose) polymerase (PARP) [17]. The Food and Drug Administration has approved PARP inhibitors to treat metastatic prostate cancer patients with DNA repair deficiencies due to pathogenic variants in genes involved in homologous recombination repair (HRR), including *BARD1*. The phase 2 LYNK-002 trial of Olaparib for patients with mutations in HRR or homologous recombination deficiency (HRD)-positive advanced breast carcinoma, malignant solid tumors, and ovarian carcinoma is ongoing [18]. With the widespread use of multiple gene mutation screenings, numerous *BARD1* pathogenic variants have been identified in breast and ovarian cancer patients to be considered for PARPi treatment. Moreover, the prevalence and frequency of *BARD1* mutations vary across different ethnic groups in breast cancer. Therefore, understanding the cancer risk and phenotypic presentations of *BARD1* mutations in Chinese breast cancer patients can contribute to informed clinical management decisions.

## 2. Materials and Methods

### 2.1. Participants and Selection Criteria

A cohort of 2658 patients with breast and ovarian cancers was recruited by the Hong Kong Hereditary Breast Cancer Family Registry on the following criteria: (1) at least one first- or second-degree relative with *BRCA*-associated cancer, regardless of age; (2) diagnosis of breast cancer at age 45 or younger; (3) bilateral breast cancer; (4) triple-negative breast cancer; (5) cancers with medullary-type histology; (6) belonging to a *BRCA* mutation-related family; (7) male breast cancer; (8) ovarian cancer. Medical personnel obtained clinicopathologic characteristics of the patients from their medical records (Table 1). To validate the performance characteristics of next-generation sequencing (NGS) and evaluate its accuracy, known *BRCA1/2*-positive control and anonymous normal local negative control individuals were included [2]. Patients carrying PVs in our 30 genes panel were excluded from our analysis.

### 2.2. Multi-Gene Panel Testing by NGS

Genomic DNA extracted from the peripheral blood underwent multi-gene sequencing analysis utilizing next-generation sequencing (NGS). Library preparation, sequencing, bioinformatics, variant interpretation, annotation, and a statistical analysis were conducted as previously outlined [2]. Paired sequencing reads were aligned to the human reference genome sequence GRCh37/hg19. Variants with a minor allele frequency of at least 1%, as reported by the 1000 Genomes Project [19], were excluded from manual variant curation. The *BARD1* reference transcript accession (NM_000465.3) and variant nomenclature adhere to the Human Genome Variation Society (HGVS) guidelines and were verified using LUMC Mutalyzer 3 (http://mutalyzer.nl (30 May 2025)).

### 2.3. Statistical Analysis

Fisher’s exact test was employed to investigate the association between selection variables and mutation status. The significance threshold for all analyses was established at a *p*-value of < 0.05. Data analyses were performed using the statistical software R (version 3.4.2) [20].

## 3. Result

### 3.1. Patients’ Characteristics of the Cohort

Our testing cohort comprised 2658 breast and ovarian cancer patients. The median age at diagnosis of breast cancer was 43 years (range 18–90), and the median age at diagnosis of ovarian cancer was 47.5 years (range 9–85). Among these patients, 2318 (87.2%) were diagnosed with breast cancer and 199 (7.5%) with ovarian cancer, while 141 (5.3%) were diagnosed with both breast and ovarian cancers. Bilateral breast cancers were observed in 568 patients (23.1%). The majority of breast cancers were classified as ductal carcinoma (NOS type) (2144; 72.7%). A significant proportion of breast cancers were of the luminal A subtype (1263; 56.9%), followed by triple-negative breast cancers (TNBC) (506; 22.8%). Most of the breast tumors were diagnosed at early stages (0, I, or II) (2250; 86.1%), and grading favored grades 2 or 3 (906, 43.8% and 822, 39.7%, respectively). Most ovarian cancers were diagnosed as epithelial cancers (285; 96.3%), and the majority were of high grade (189; 68%). A positive family history of breast cancer (in first- or second-degree relatives) was observed in 1072 patients (40.3%). Family histories of ovarian cancer and prostate cancer were recorded in 189 (7.1%) and 145 (5.5%) of their patient’s relatives, respectively. Comprehensive clinicopathological characteristics are shown in Table 1.

### 3.2. Characteristics of BARD1 Mutation Carriers

Heterozygous pathogenic mutations in *BARD1* were identified in 12 probands, resulting in a mutation frequency rates of 0.45% among high-risk breast cancer patients and 0.29% among ovarian cancer patients, while none of the *BARD1* carrier had double heterozygous mutations. Eleven of the mutation carriers had personal breast cancers (91.7%), and only one had ovarian cancer (8.3%). The median age of breast cancer diagnosis was 43 (range 24–69), while the carrier with ovarian cancer was diagnosed at age 31. All of the *BARD1* carriers were female. Only one *BARD1* carrier had multiple cancers, including ovarian cancer and cancer of the uterus. Unlike *BRCA* carriers, none of the *BARD1* carriers reported having bilateral breast cancer. Most of the breast tumors were diagnosed as invasive ductal carcinoma (NOS type) (10; 90.9%) of grade 3 (9; 100.0%). Half of the breast cancers were found to be triple-negative breast cancers (TNBC). Positive family histories of breast, colorectal, and liver cancers (in first- or second-degree relatives) were observed in four patients (33.3%). In comparison, a family history of ovarian cancer (in first- or second-degree relatives) was noted in only two patients (16.7%). Detailed pathological characteristics and family histories are shown in Table 2 and Table 3.

**Figure 1:**
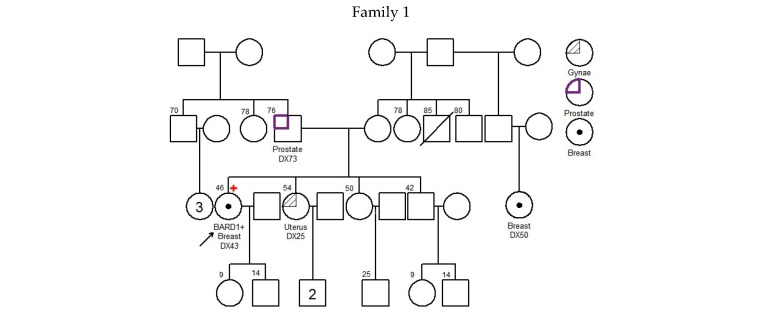

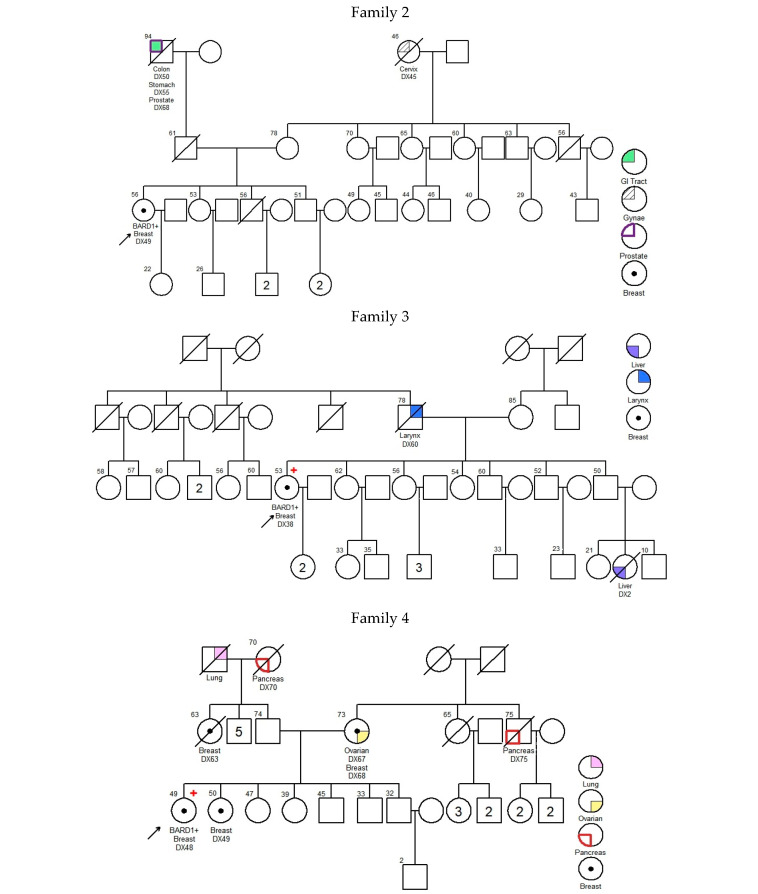

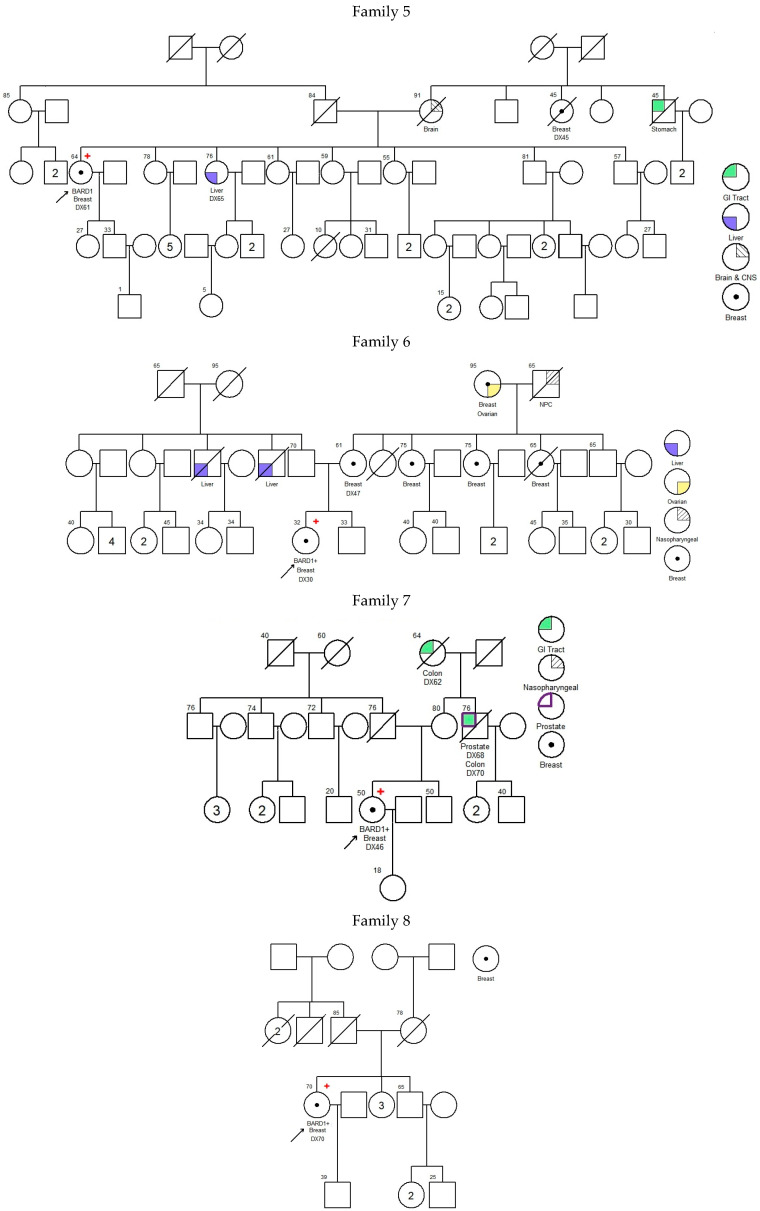

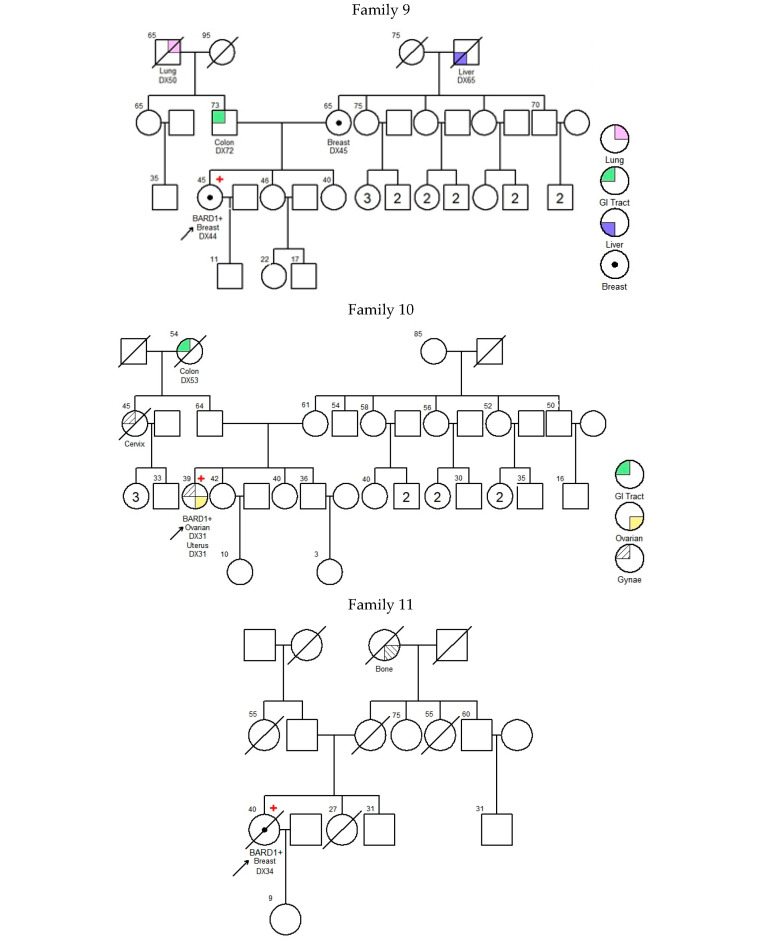

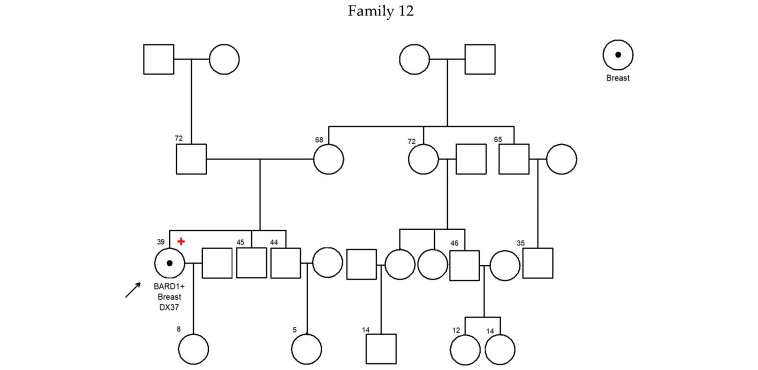
Pedigree of *BARD1* + families. Arrow refers to proband.

There was no significant difference in diagnosis age of breast cancer between *BARD1* mutation carriers, *BRCA1/2* mutation carriers, and 30 genes non-carriers. However, the histopathology of the breast cancers of the *BARD1* mutation carriers and *BRCA1/2* mutation carriers showed certain distinguishing characteristics. Interestingly, all *BARD1* and *BRCA1* mutation carriers were female, whereas 2.8% of *BRCA2* mutation carriers were male (Table 2). Unlike *BRCA1/2* mutation carriers, bilateral breast cancer was not commonly seen in *BARD1* mutation carriers, while 29.8% of the *BRCA1* and *BRCA2* mutation carriers developed bilateral breast cancers (*p*-value = 0.039). *BARD1* carriers favor the development of high-grade invasion breast cancer, the same as *BRCA1* carriers (*p*-value = 0.204), while *BRCA2* carriers and non-carriers develops less aggressive tumors (*p*-values = 0.004 and <0.001 respectively). *BARD1* and *BRCA1/2* mutation carriers had a strong family history of breast cancer. A significant increase in the family history of liver cancer was observed in *BARD1*-mutated families compared to *BRCA1*-mutated families; 33% of *BARD1* mutation carriers had a family history of liver cancer, whereas only 11.6% and 11.3% of *BRCA1* mutation carriers and non-carriers did, respectively (*p*-value = 0.049 and 0.04). There was no significant difference between *BARD1* and *BRCA2* mutation carriers (*p*-value = 0.09). A similar observation was made regarding the family history of prostate cancer, which was present in 25% of *BARD1* carriers compared to only 2.4% of *BRCA1* carriers (*p*-value = 0.005). No significant difference was found between *BARD1* and *BRCA2* carriers, confirming the higher chance of having prostate cancer for *BRCA2* carriers (*p*-value = 0.216). *BARD1* carriers had significant family histories of liver, prostate, and cervical cancers compared to non-carriers (*p*-values = 0.04, 0.018, and 0.037, respectively). Additionally, *BARD1* mutations were associated with a higher grade of disease compared to *BRCA2* carriers and mutation-free non-carriers (*p*-values = 0.004 and <0.001, respectively; Table 2). The associations of age at ovarian cancer diagnosis and histology between *BARD1* mutation carriers and *BRCA1/2* mutation carriers were not calculated due to limited case numbers. Details of family histories of *BARD1* mutation carriers are shown in Figure 1 and Table 3.

### 3.3. Mutation Spectrum in BARD1

**Figure 2:**
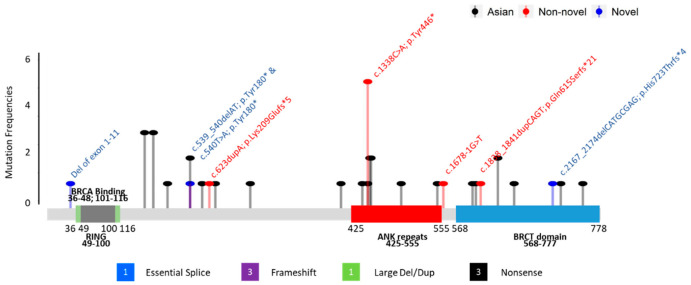
Mutations found in *BARD1.* The protein domains RING (Grey), BRCA binding (green), ankyrin (ANK, red), and BRCA1 carboxy-terminal (BRCT, blue) are indicated; 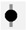
—Asian mutation; 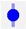
—novel mutation; 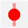
—recurrent mutation.

In our cohort, 8 unique mutations were identified from 12 probands. Four (50%) of the mutations were nonsense mutations, two (25%) resulted in frameshifts and early termination, one (12.5%) occurred at splice sites, and one (12.5%) was a large deletion of exons 1–11. Three novel mutations were identified: c.540T>A; p.(Tyr180*), c.2167_2174delCATGCGAG; p.(His723Thrfs*4), and deletion of exons 1–11. There was no specific genomic regional clustering for these mutations in *BARD1*. The most frequent mutation, c.1338C>A; p.(Tyr446*) was seen in 5 (41.7%) unrelated families. This variant is located in exon 5, within the repeated domains of ANK. Details of family histories and distribution on the functional domains of these mutation variants are shown in Figure 2 and Table 3.

## 4. Discussion

Case–control studies have found a low to moderate association between breast cancer and pathogenic and likely pathogenic (P/LP) variants in the *BARD1* gene, with a prevalence range of 0.1% to 0.51% in patients with breast cancer [1,3,28]. Recently, the absolute risk range for breast cancer in *BARD1* carriers has been revised from 15–40% to 20–40% in the NCCN Genetic/Familial High-Risk Assessment 2023.3 Guidelines. *BARD1* PVs in breast cancer patients of European ancestry had an odds ratio (OR) of 2.2 (*p*-value = 0.002; n = 28,536) [1]. Another study found an OR of 3.2 (*p*-value = 0.012; n = 2127) for breast cancer patients with a family history of breast cancer, while a large-scale case–control study indicated an OR of 2.3 (*p*-value = 0.04; n = 13,935) [3,29], showing that *BARD1* is associated with low to moderate risk for breast cancer. In a retrospective study of approximately 48,700 breast cancer cases and 20,800 ovarian cancer cases, *BARD1* was identified as a moderate-risk gene for breast cancer (OR = 2.90, 95% CIs: 2.25–3.75, *p*-value < 0.0001) but not for ovarian cancer (OR = 1.36, 95% CIs: 0.87–2.11, *p*-value = 0.1733) [30]. Moreover, a stronger association (OR = 5.4; *p*-value < 0.00001; n = 4469) between *BARD1* PVs and familial breast cancer patients was reported. The risk was further enhanced (OR = 12.0; *p*-value < 0.00001; n = 782) for breast cancer patients who diagnosed under 40 years of age, suggesting that *BARD1* may be a risk gene for early-onset familial breast cancer [28]. However, the association of *BARD1* with ovarian cancer has not been convincingly established [1,22,30]. In a meta-analysis, the mutation frequency rates in the *BARD1* gene among breast cancer and ovarian cancer patients from mixed populations were 0.25% and 0.12%, respectively [30]. In another study including data from Australia, USA, and UK, the prevalence of PVs in *BARD1* in the breast cancer group was only 0.12% [22]. In our Asian cohort, the prevalence rates of PVs in *BARD1* in the breast cancer group (0.45%) and the ovarian cancer group (0.29%) were significantly higher than the reported mutation frequencies. However, the breast cancer risk estimates of *BARD1* PVs for Caucasians and Asians show no substantial difference and the frequency of *BARD1* mutations in general population controls (from mixed populations) is 0.09% [30].

Individuals with *BARD1* and *BRCA1* germline pathogenic mutations have been found to have a higher incidence rates of aggressive breast cancer phenotypes, such as TNBCs, which are associated with higher rates of recurrence, progression, and mortality [31,32,33]. At the molecular and protein levels, *BARD1* shows a significant degree of structural and functional similarity to *BRCA1*, and breast cancers occurring in individuals with *BARD1* germline PVs exhibit a similar somatic gene expression profile to those with *BRCA1* pathogenic variants. An example of this is when a patient with breast cancer had a germline *BARD1* deletion or loss of heterozygosity in the tumor, resulting in a basal-like gene expression profile similar to those observed in cancers associated with *BRCA1* germline PVs [31,34]. No significant differences in clinicopathological characteristics were observed in our Chinese cohort between *BARD1* and *BRCA1* mutation carriers, except that carriers of the *BRCA1* mutation had a higher incidence of bilateral breast cancer. However, *BARD1* mutation carriers with bilateral breast cancer have been reported in Polish and Belarusian populations [29] but not in Asian populations. Significant differences in molecular subtypes and grading were observed between *BARD1* and *BRCA2* mutation carriers. Among *BRCA2* mutation carriers, 84.5% had a preference for being ER-positive with a molecular subtype of luminal A (71%), while only 54.5% of *BARD1* mutation carriers developed ER-positive breast cancer (*p*-value = 0.022), with 30% in the molecular subtype of luminal A. In our *BARD1* mutation carriers, we observed that 50% of them harbored TNBC, a frequency similar to that of *BRCA1* mutation carriers (58.2%). In comparison, only 11.6% of *BRCA2* mutation carriers were TNBC (*p*-value = 0.005), and 18.6% of mutation-negative patients were TNBC (*p*-value = 0.025). This association aligns with studies in the Spanish population and other European studies [35,36]. The Breast Cancer Association Consortium and the CARRIERS case–control studies also found associations between *BARD1* PVs and an increased risk of triple-negative breast cancer [37,38]. All of our *BARD1* mutation carriers developed high-grade breast cancer, while only 44.7% of *BRCA2* mutation carriers (*p*-value = 0.004) and 39.7% of mutation-negative patients (*p*-value < 0.001) developed high-grade breast cancer. Another Asian study from Singapore also found that patients with *BARD1* PVs developed more aggressive triple-negative breast cancer and high-grade breast cancers [39].

Germline copy number variants (CNVs) in the *BRCA1* and *BRCA2* genes account for less than 5% of known pathogenic variants in these genes [40]. CNVs in the *BARD1* gene have also been observed. Deletions of exon 1 [41] and exon 2 [42] have been reported in breast cancer patients, while deletions of exons 8 to 11 and the entire gene were identified in ovarian cancer patients [43]. Additionally, a deletion of exons 8 to 11 in the *BARD1* gene has also been identified in a family with hereditary colorectal cancer syndrome [44]. Our cohort identified a deletion of the entire *BARD1* gene from exons 1 to 11. Two CNVs (deletion of exons 4 to 11 and duplication of exons 1 to 9) in the *BARD1* gene were also identified among non-cancer controls [23]. CNVs in *BARD1* are not rare events; they accounted for at least 8.3% of known PVs in our cohort.

Two regions in the *BARD1* gene have been reported to have an increased density of pathogenic variants [30]. The first overlaps with the RING-finger domain, extending from exon 2 to around 230 amino acids in exon 4. The second “hotspot” region extends from exon 5 to exon 10, covering the ANK repeat and the BRCT domains. However, unlike the *BRCA1* and *BRCA2* genes, which have clustered regions associated with breast and ovarian cancers, no clear hotspot could be identified in *BARD1*. All mutations identified in our cohort, except for the CNV mentioned earlier, were located in ANK repeat and BRCT domains (see Figure 2). Among the 12 *BARD1* mutation carriers we identified, five carried a c.1338C>A; p.(Tyr446*) mutation in exon 5, which is located in the repeated domains of ANK. This variant has also been reported in unselected breast and colorectal cancer patients in the Chinese population [23,24], and there were two submissions from Ambry Genetics and Invitae in ClinVar. Excluding the above-mentioned patients from China, this c.1338C>A; p.(Tyr446*) mutation was not reported in a large cumulative summary of the *BARD1* PVs mutation spectrum identified from breast and ovarian cancer patients, with the entire *BARD1* coding sequence sequenced [30]. In the gnomAD control, this variant was also seen once in the East Asian and European (non-Finnish) populations. These findings demonstrate that although the mutation is shared across ethnicities, it is not a common recurrent mutation; however, the frequency of its identification in the Chinese population is relatively higher (Table 2 and Supplementary Table S1).

Many studies have widely discussed the association of the *BARD1* missense mutation c.1670G>C; p.(Cys557Ser) to breast cancer risk. Extensive studies in Iceland, Finland, Latin or South America, and Italy showed this germline variant was associated with an increase of two- to four-fold in breast cancer risk [26,27]. In contrast, other reports from Yoruba, Chinese, Japanese, Australian, and African American individuals did not show similar findings [45]. A meta-analysis also found no evidence to support this association, except in women with a strong family history, where these carriers had a 3.4-fold increase in breast cancer risk [46]. The association between familial breast cancer susceptibility on this missense variant remains controversial. However, none of our high-risk patients carried this missense variant, further impeding our understanding of the clinical relevance of *BARD1*.

*BARD1* mutation carriers in our study were more likely to have a family history of liver, prostate, and cervical cancers than patients who tested negative for the 30 gene panel (*p*-values = 0.04, 0.018, and 0.037, respectively). In our study, no significant difference was found in the family histories of breast cancer between *BARD1* mutated carriers and non-carriers, largely due to selection bias, as ‘family history of breast cancer’ is one of our recruiting criteria. Current NCCN surveillance management guidelines recommend only annual breast screening starting at age 40 for *BARD1* mutation carriers but no surveillance management for other related cancers. However, *BARD1* PVs have been identified in patients with not only breast cancer but also in patients with neuroblastoma, colon cancer, liver cancer, lung cancer, and acute myeloid leukemia [21]. *BARD1* has also be found as prognosis-related genes of liver cancer and used for predicting the survival of liver cancer patients [47]. For prostate cancer, a study from Poland confirmed *BARD1* mutation carriers were not at elevated risk of prostate cancer [48]; however, another study of 9185 men with aggressive prostate cancer from 18 international studies provided evidence of greater risk (OR ≥ 2) but the carrier frequency differences between aggressive and non-aggressive prostate cancer were not statistically significant [49]. These variants may confer low to moderate penetrance effects, which still require more evidence and convincing risk assessments for recommendations on surveillance for carriers of *BARD1* pathogenic variants concerning other cancers.

## 5. Conclusions

We demonstrated that the mutation frequency rates of *BARD1* were 0.45% among high-risk breast cancer patients and 0.29% among ovarian cancer patients. We identified three novel mutations and a recurrent mutation in the *BARD1* gene. Half of the *BARD1* mutation carriers were found to have TNBC and were likely to have familial aggregation of liver, prostate, and cervical cancers compared to patients who tested negative for mutations in the 30 gene panel. Mutation screening for *BARD1* should be included in the test panel for breast cancer patients. However, more comprehensive surveillance management may be considered, even given the low penetrance of *BARD1*, especially for Asian patients. More clinical evidence is needed to demonstrate the effectiveness of PARP inhibitors in patients with *BARD1* mutations.

## Figures and Tables

**Table 1:** Clinicopathologic characteristics of study cohort.

		N = 2658	
		N	%
**Gender**	F	2584	97.2%
	M	74	2.8%
**Personal cancer**	Breast cancer	2318	87.2%
	Breast cancer and OV cancers	141	5.3%
	OV cancers	199	7.5%
**Multiple cancers**	Yes	352	13.2%
	No	2306	86.8%
**1st dx age (breast cancer)**	Mean	44.9	
	Median	43	
	SD	11.4	
	Range	18–90	
**1st dx age (ovarian cancer)**	Mean	47.3	
	Median	47.5	
	SD	12.6	
	Range	9–85	
**Bilateral breast cancers**	Yes	568	23.1%
	No	1891	76.9%
**Personal other cancers**	Uterus cancer	76	2.9%
**Family history of cancers** **(in 1st and 2nd degree)**	Breast cancer	1072	40.3%
	Ovarian cancer	189	7.1%
	Colorectal cancer	488	18.4%
	Liver cancer	312	11.7%
	Prostate cancer	145	5.5%
	Cervical cancer	76	2.9%
	Stomach cancer	250	9.4%
	Lung cancer	542	20.4%
**Breast cancer**		**N = 3027**	
**Histology**	Ductal	2144	72.7%
	In situ	492	16.7%
	Others	314	10.6%
	Not stated	77	
**Grade (invasive grade)**	1	340	16.4%
	2	906	43.8%
	3	822	39.7%
	Not stated	467	
**Molecular subtype** **(invasive tumor only)**	TNBC	506	22.8%
	Her2	180	8.1%
	Luminal A	1263	56.9%
	Luminal B	270	12.2%
	Unclassified/Not stated	316	
**Stage**	0	530	18.6%
	I	1062	37.3%
	II	858	30.1%
	III	296	10.4%
	IV	100	3.5%
	NS	181	
**Ovarian cancers**		**N = 340**	
**Main site**	Ovarian	271	81.1%
	Fallopian tube	15	4.5%
	Peritoneal	19	5.7%
	Uterus	23	6.9%
	Mixed	6	1.8%
	NS	6	
**Histological type**	Epithelial	285	96.3%
	Germ Cell	5	1.7%
	Stromal	4	1.4%
	Others	0	0.0%
	Mixed	2	0.7%
	NS	44	0.0%
**Grade**	1	33	11.9%
	2	54	19.4%
	3	189	68.0%
	Mixed	2	0.7%
	NS	62	
**Stage**	1	108	36.9%
	2	34	11.6%
	3	114	38.9%
	4	37	12.6%
	NS	47	

**Table 2:** Characteristics of *BARD1* carriers comparing with BRCA1/2 carriers and mutation negative patients.

	*BARD1*+ N = 12		*BRCA1*+ N = 251		*BRCA2*+ N = 259		*BRCA1/2*+ N = 510		Negative N = 2136		Total N = 2658		*p*-Value			
													*BARD1*+ vs. *BRCA1*+	*BARD1*+ vs. *BRCA2*+	*BARD1*+ vs. *BRCA1/2*+	*BARD1*+ vs. Negative
	N	%	N	%	N	%	N	%	N	%	N	%				
**Gender**																
F	12	100.0%	251	100.0%	244	94.2%	495	97.1%	2077	97.2%	2584	97.2%	1	1	1	1
M	0	0.0%	0	0.0%	15	5.8%	15	2.9%	59	2.8%	74	2.8%				
**Personal Cancer**																
Breast	11	91.7%	142	56.6%	207	79.9%	349	68.4%	1958	91.7%	2318	87.2%	0.058	0.851	0.313	0.649
Breast and ovarian	0	0.0%	45	17.9%	26	10.0%	71	13.9%	70	3.3%	141	5.3%				
Ovarian	1	8.3%	64	25.5%	26	10.0%	90	17.6%	108	5.1%	199	7.5%				
**Personal Multiple Cancer**																
Y	1	8.3%	53	21.1%	40	15.4%	93	18.2%	258	12.1%	352	13.2%	0.469	1	0.703	1
N	11	91.7%	198	78.9%	219	84.6%	417	81.8%	1878	87.9%	2306	86.8%				
**Breast CA 1st Dx Age**																
Mean	45.364		40.893		43.15		42.145		45.464		44.897		0.241	0.55	0.387	0.978
Median	43		40		42		41		43		43		0.211	0.679	0.424	0.986
SD	11.716		9.712		9.27		9.525		11.729		11.447					
Range	30–70		22–73		21–73		21–73		18–90		18–90					
**Ovarian CA 1st Dx Age**																
Mean	31		51.743		55.462		52.944		42.354		47.335		NA	NA	NA	NA
Median	31		51		55.5		52		43		47.5		NA	NA	NA	NA
SD	NA		10.481		9.348		10.25		12.347		12.567					
Range	31–31		17–85		31–75		17–85		9–74		9–85					
**Bilateral cancers**																
Y	0	0.0%	62	33.2%	63	27.0%	125	29.8%	443	21.8%	568	23.1%	0.019	0.071	0.039	0.136
N	11	100.0%	125	66.8%	170	73.0%	295	70.2%	1585	78.2%	1891	76.9%				
**Personal other cancers**																
Uterus cancer	1	8.3%	4	1.6%	4	1.5%	8	1.6%	67	3.1%	76	2.9%	0.21	0.204	0.19	0.321
**Family history of cancers (in 1st and 2nd deg)**																
Breast cancer	4	33.3%	135	53.8%	164	63.3%	299	58.6%	769	36.0%	1072	40.3%	0.237	0.063	0.136	1
Ovarian cancer	2	16.7%	68	27.1%	28	10.8%	96	18.8%	91	4.3%	189	7.1%	0.525	0.629	1	0.092
Colorectal cancer	4	33.3%	47	18.7%	52	20.1%	99	19.4%	385	18.0%	488	18.4%	0.256	0.278	0.266	0.248
Liver cancer	4	33.3%	29	11.6%	37	14.3%	66	12.9%	242	11.3%	312	11.7%	0.049	0.09	0.063	0.04
Prostate cancer	3	25.0%	6	2.4%	34	13.1%	40	7.8%	102	4.8%	145	5.5%	0.005	0.216	0.068	0.018
Cervical cancer	2	16.7%	8	3.2%	12	4.6%	20	3.9%	54	2.5%	76	2.9%	0.07	0.122	0.087	0.037
Stomach cancer	2	16.7%	38	15.1%	32	12.4%	70	13.7%	178	8.3%	250	9.4%	1	0.651	0.675	0.266
Lung cancer	2	16.7%	60	23.9%	72	27.8%	132	25.9%	408	19.1%	542	20.4%	0.737	0.522	0.739	1
**Breast cancer**	**N = 11**		**N = 249**		**N = 296**		**N = 545**		**N = 2471**		**N = 3027**					
**Histology**																
Ductal	10	90.9%	201	83.8%	208	72.5%	409	77.6%	1725	71.5%	2144	72.7%	0.378	0.677	0.856	0.619
In situ	1	9.1%	12	5.0%	48	16.7%	60	11.4%	431	17.9%	492	16.7%				
Others	0	0.0%	27	11.2%	31	10.8%	58	11.0%	256	10.6%	314	10.6%				
NS	0		9		9		18		59		77					
**Molecular subtype**																
TNBC	5	50.0%	121	61.1%	27	12.9%	148	36.3%	353	19.6%	506	22.8%	0.106	0.012	0.25	0.053
Her2	0	0.0%	6	3.0%	6	2.9%	12	2.9%	168	9.3%	180	8.1%				
Luminal A	3	30.0%	66	33.3%	149	71.0%	215	52.7%	1045	58.0%	1263	56.9%				
Luminal B	2	20.0%	5	2.5%	28	13.3%	33	8.1%	235	13.0%	270	12.2%				
Unclassified/NS	0		39		38		77		239		316					
**TNBC**																
Y	5	50.0%	121	58.2%	27	11.6%	148	33.6%	353	18.6%	506	21.5%	0.746	0.005	0.318	0.025
N	5	50.0%	87	41.8%	206	88.4%	293	66.4%	1548	81.4%	1846	78.5%				
**Grade (invasive grade)**																
1	0	0.0%	6	3.2%	12	6.3%	18	4.8%	322	19.1%	340	16.4%	0.204	0.004	0.029	0
2	0	0.0%	47	25.1%	93	48.9%	140	37.1%	766	45.5%	906	43.8%				
3	9	100.0%	134	71.7%	85	44.7%	219	58.1%	594	35.3%	822	39.7%				
NS	1		50		58		108		358		467					
**OV cancers**	**N = 1**		**N = 109**		**N = 52**		**N = 161**		**N = 178**		**N = 340**					
**Site**																
Ovarian	1	100.0%	82	75.9%	38	76.0%	120	75.9%	150	85.7%	271	81.1%	1	1	1	1
Fallopian tube	0	0.0%	11	10.2%	3	6.0%	14	8.9%	1	0.6%	15	4.5%				
Peritoneal	0	0.0%	11	10.2%	6	12.0%	17	10.8%	2	1.1%	19	5.7%				
Uterus	0	0.0%	1	0.9%	2	4.0%	3	1.9%	20	11.4%	23	6.9%				
Mixed	0	0.0%	3	2.8%	1	2.0%	4	2.5%	2	1.1%	6	1.8%				
NS	0		1		2		3		3		6					
**Histological Type**																
Epithelial	1	100.0%	101	100.0%	44	97.8%	145	99.3%	139	93.3%	285	96.3%	1	1	1	1
Germ Cell	0	0.0%	0	0.0%	0	0.0%	0	0.0%	5	3.4%	5	1.7%				
Stromal	0	0.0%	0	0.0%	0	0.0%	0	0.0%	4	2.7%	4	1.4%				
Others	0	0.0%	0	0.0%	0	0.0%	0	0.0%	0	0.0%	0	0.0%				
Mixed	0	0.0%	0	0.0%	1	2.2%	1	0.7%	1	0.7%	2	0.7%				
NS	0		8		7		15		29		44					
**Grade**																
1	0	0.0%	0	0.0%	1	2.4%	1	0.7%	32	23.4%	33	11.9%	1	1	1	1
2	0	0.0%	2	2.0%	2	4.9%	4	2.9%	50	36.5%	54	19.4%				
3	1	100.0%	97	98.0%	37	90.2%	134	95.7%	54	39.4%	189	68.0%				
Mixed	0	0.0%	0	0.0%	1	2.4%	1	0.7%	1	0.7%	2	0.7%				
NS	0		10		11		21		41		62					
**Stage**																
1	0	0.0%	9	8.9%	8	17.0%	17	11.5%	91	63.2%	108	36.9%	0.196	0.063	0.087	0.2
2	1	100.0%	10	9.9%	2	4.3%	12	8.1%	21	14.6%	34	11.6%				
3	0	0.0%	62	61.4%	27	57.4%	89	60.1%	25	17.4%	114	38.9%				
4	0	0.0%	20	19.8%	10	21.3%	30	20.3%	7	4.9%	37	12.6%				
NS	0		8		5		13		34		47					

**Table 3:** Germline heterozygous mutations identified in *BARD1*.

Family	Variants	Exon/ Intron	Domain	Personal History		Pathology				1st and 2nd Family History		Other Mutations	Novel/ Reference
				Dx	Cancer	Histology	ER	PR	Her2	Dx	Cancer		
001	c.539_540delAT; p.Tyr180*	exon 4		43	Breast	IDC	+	+	+	25 73	Uterus Prostate	--	[21]
002	c.540T>A; p.Tyr180*	exon 4		49	Breast	IDC	−	−	−	45 50 55 68	Cervix Colon, Stomach, Prostate	--	Novel
003	c.623dupA; p.Lys209Glufs*5	exon 4		38	Breast	IDC	+	+	−	60 2	Larynx Liver	*BARD1* (VUS): c.1570A>G; p.Asn524Asp	[22]
004	c.1338C>A; p.Tyr446*	exon 5	Ankyrin	48	Breast	IDC	−	−	−	49 63 68 67 70 75 UK	Breast Breast Breast and Ovarian Pancreas Pancreas Lung	BMPR1A (VUS): c.910C>A; p.Gln304Lys	[23,24]
005	c.1338C>A; p.Tyr446*	exon 5	Ankyrin	61	Breast	IDC	−	−	−	UK 45 65 UK	Brain Breast Liver Stomach	--	[23,24]
006	c.1338C>A; p.Tyr446*	exon 5	Ankyrin	30	Breast	IDC	−	−	−	47 UK UK UK UK UK UK UK	Breast Breast Breast Breast Breast, Ovarian NPC Liver Liver	--	[23,24]
007	c.1338C>A; p.Tyr446*	exon 5	Ankyrin	46	Breast	IDC	−	−	−	62 70 68	Colon Colon, Prostate	--	[23,24]
008	c.1338C>A; p.Tyr446*	exon 5	Ankyrin	70	Breast	Papillary	+	+	−	80	Breast	MLH1 (VUS): c.1730C>T; p.Ser577Leu MSH6 (VUS): c.3257C>G; p.Pro1086Arg	[23,24]
009	c.1678-1G>T	intron 7		44	Breast	IDC	−	−	−	45 72 65 50	Breast Colon Liver Lung	--	[25]
010	c.1838_1841dupCAGT; p.Gln615Serfs*21	exon 9	BRCT repeats	31 31	Ovarian Uterus	Endometrioid Endometrioid	NA	NA	NA	45 54	Cervix Colon	MUTYH (VUS): c.934-2A>G (heterozygous)	[26,27]
011	c.2167_2174delCATGCGAG; p.His723Thrfs*4	exon 10	BRCT repeats	34	Breast	IDC	+	+	−	UK	Bone	--	Novel
012	deletion of whole gene (exons 1–11)	exons 1–11	NA	37	Breast	IDC	+	+	FISH Equivocal	No Family History of Cancer		ATM (VUS): c.6154G>A; p.Glu2052Lys	Novel

## Data Availability

The dataset supporting the conclusions of this article is included within the article.

## Supplementary Materials

The following supporting information can be downloaded at: https://www.mdpi.com/article/10.3390/cancers17152524/s1, Table S1. BARD1 pathogenic variants identified from breast or ovarian cancer patients from Asian populations.
