# Supplementary figures and images for: Germline BARD1 Mutation in High-Risk Chinese Breast and Ovarian Cancer Patients
Source: Cancers (Basel). 2025 Jul 30;17(15):2524. doi: 10.3390/cancers17152524 (PMC12346768; doi:10.3390/cancers17152524)

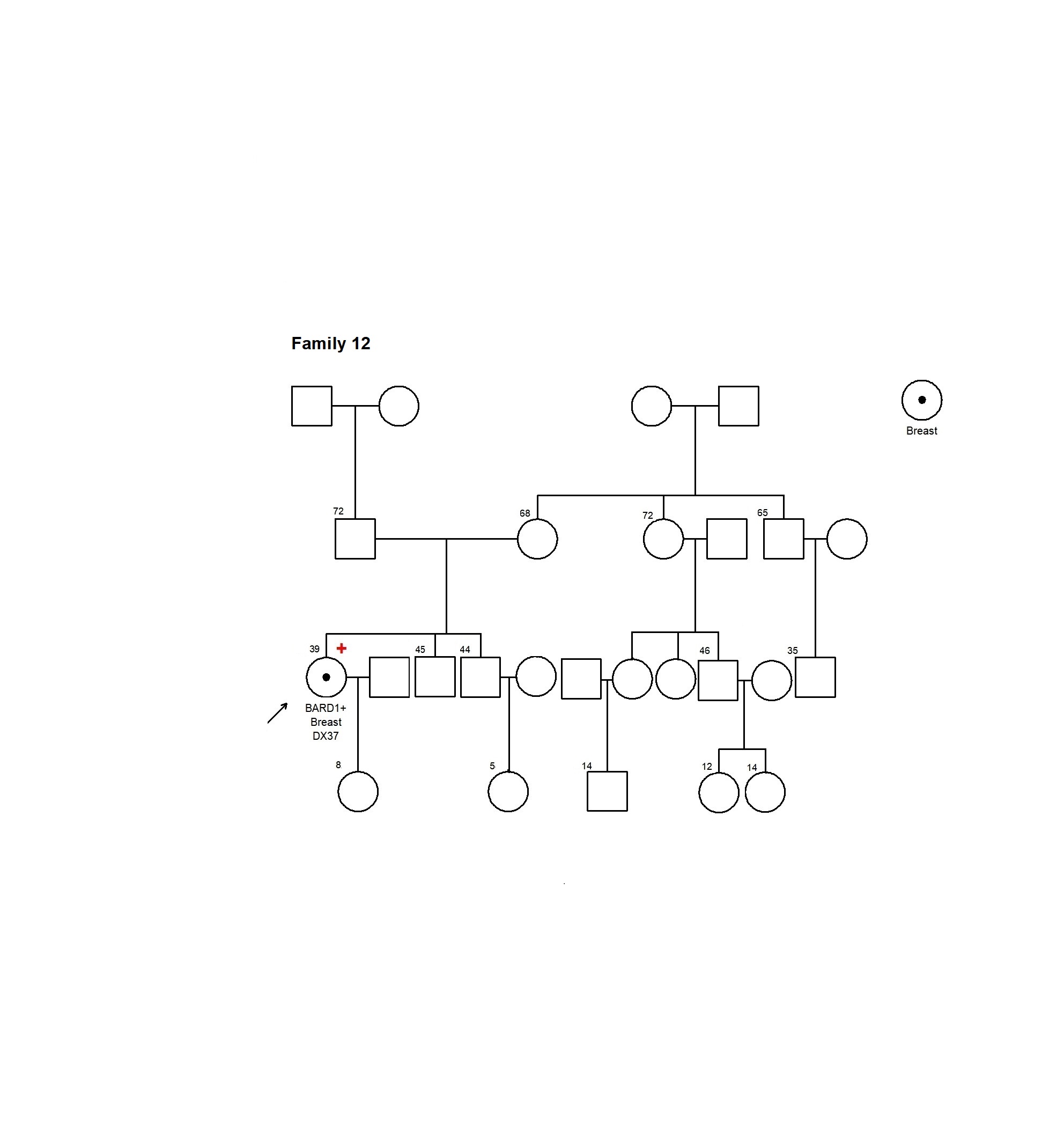

Supplement: Supplementary file 1 [file cancers-17-02524-s001.zip › cancers-3758610-figures.jpg]
